# Effect of Admission Hyperglycemia on Short-Term Prognosis of Patients with Non-ST Elevation Acute Coronary Syndrome without Diabetes Mellitus

**DOI:** 10.1155/2021/1321289

**Published:** 2021-12-06

**Authors:** Wei Liu, Zhijuan Li, Shiying Xing, Yanwei Xu

**Affiliations:** ^1^Department of Cardiology, The First Affiliated Hospital of Henan University of Science and Technology, Luoyang 471003, China; ^2^School of Mechatronics Engineering, Henan University of Science and Technology, Luoyang 471003, China

## Abstract

**Objective:**

To evaluate the effect of admission hyperglycemia on the short-term prognosis of patients with non-ST elevation acute coronary syndrome (NSTE-ACS) without diabetes mellitus.

**Methods:**

The clinical data of 498 patients with NSTE-ACS admitted to the Department of Cardiology of the First Affiliated Hospital of Henan University of Science and Technology between March 2018 and November 2020 were analyzed. Based on the blood glucose (BG) level at admission, patients were divided into three groups: A (BG < 7.8 mmol/L), B (7.8 mmol/L ≤ BG < 11.1 mmol/L), and C (BG ≥ 11.1 mmol/L). The clinical data of the three groups were compared.

**Results:**

There was no significant difference between the three groups in terms of age, sex, hypertension, hyperlipidemia, smoking, and history of myocardial infarction (*p* > 0.05). However, there were significant differences in the incidences of multivessel disease, renal insufficiency, pump failure, and emergency percutaneous coronary intervention, and levels of high-sensitivity C-reactive protein, cardiac troponin T, and creatine kinase isoenzyme MB among the three groups (*p* < 0.05 for all). The incidences of severe pump failure, malignant arrhythmias, and death were significantly higher in groups B and C compared to group A (*p* < 0.05). Additionally, the incidences of severe pump failure, malignant arrhythmias, and death were significantly higher in group C compared to group B (*p* < 0.05). Multivariate logistic regression analysis showed that hyperglycemia, renal insufficiency, Killip grade III/IV, and age were risk factors of in-hospital death.

**Conclusion:**

Hyperglycemia at admission is a risk factor for adverse in-hospital clinical outcomes in patients with NSTE-ACS.

## 1. Introduction

Diabetes is an important independent risk factor for coronary atherosclerosis. Many previous studies have confirmed that hyperglycemia at admission is common in patients with acute coronary syndrome (ACS), and it is a risk factor for in-hospital death and complications [1–4]. Previous epidemiological studies showed that 25–50% of ACS patients had elevated blood glucose (BG) level at admission. Recent studies suggest that the effects of hyperglycemia on the prognosis of ACS differ between diagnosed and undiagnosed diabetes. Hyperglycemia is a stronger predictor of adverse events in ACS patients without known diabetes than those with history of diabetes [5, 6]. Although there is a clear correlation between hyperglycemia and the occurrence and development of cardiovascular disease, the significance and treatment of hyperglycemia in ACS patients are not clear. The aim of this study was to investigate the effect of admission hyperglycemia on the short-term prognosis in patients with the non-ST elevation ACS (NSTE-ACS) without diabetes.

## 2. Data and Methods

### 2.1. Research Populations

**Figure 1:**
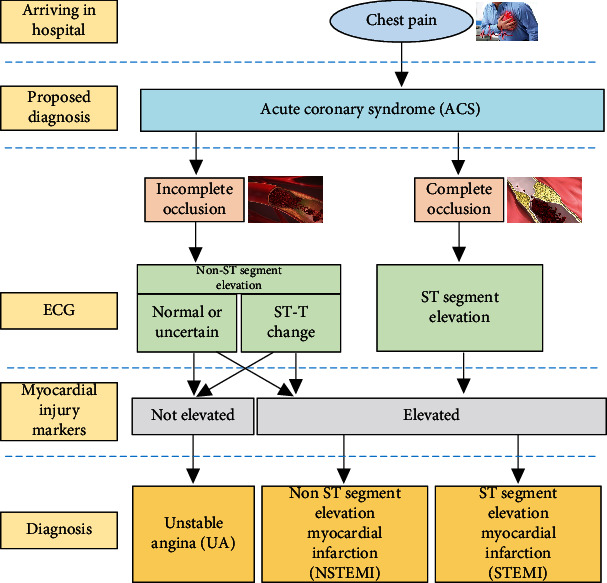
Classification of the acute coronary syndrome.

ACS is a common, serious heart disease that comprises a group of syndromes due to rupture or invasion of the coronary atherosclerotic plaque and secondary complete or incomplete occlusive thrombosis. ACS includes acute ST-segment elevation myocardial infarction, acute non-ST-segment elevation myocardial infarction, and unstable angina (UA). The clinical classification of the acute coronary syndrome is shown in Figure 1.

This was a retrospective analysis of prospectively enrolled consecutive NSTE-ACS patients treated at the Department of Cardiology of the First Affiliated Hospital of Henan University of Science and Technology from March 2018 to November 2020. NSTE-ACS was diagnosed on the basis of typical angina symptoms lasting for >10 minutes, accompanied by at least one of the following: ST-segment depression ≥ 0.5 mm, 0.5–1.0 mm transient ST-segment elevation in two consecutive leads for <30 minutes, T-wave inversion > 1 mm before the chest pain or within 12 hours after the chest pain, and/or myocardial enzymes (cardiac troponin T (TnT) or creatine kinase isozymes MB (CK-MB)) exceeding the upper limit of the normal values. Patients were excluded if they had past diabetes, incomplete clinical and coronary angiography data, admission BG level > 20 mmol/L, symptoms of ketoacidosis, and/or new-onset diabetes. A total of 498 patients with NSTE-ACS and high BG without history of diabetes were enrolled. The Ethical Committee of the First Affiliated Hospital of Henan University of Science and Technology approved the study protocol.

### 2.2. Definition

Hypertension is a syndrome characterized by increased systemic arterial blood pressure (systolic pressure ≥ 140 mmHg and diastolic pressure ≥ 90 mmHg), which can be accompanied by functional or organ damage to the heart, brain, kidney, and/or other organs. Hypertension is the most common chronic disease and the main risk factor of cardiovascular and cerebrovascular diseases. Hypertension is divided into three grades according to severity: mild (systolic blood pressure: 140–159 mmHg or diastolic blood pressure: 90–99 mmHg), moderate (systolic blood pressure: 160–179 mmHg or diastolic blood pressure: 100–109 mmHg), and severe (systolic blood pressure: ≥180 mmHg or diastolic blood pressure: ≥110 mmHg).

Hyperlipidemia refers to high blood lipid level, which can cause serious diseases, such as atherosclerosis, coronary heart disease, and pancreatitis. Hyperlipidemia usually is divided into hypertriglyceridemia, hypercholesterolemia, mixed hyperlipidemia, and low high-density lipoprotein cholesterol. Hypercholesterolemia is related to age and gender of patients. The majority of triglycerides are associated with genetic and environmental changes, and metabolic disorders. Most cases of hypertriglyceridemia are usually associated with abnormal BG, and diabetes mellitus is associated with hypertriglyceridemia.

Heart failure refers to systolic and/or diastolic function of the heart, leading to impaired pumping of the blood returned from the venous circulation to the heart, causing blood stasis in the venous system, insufficient perfusion in the arterial system, and cardiac circulation disorder syndrome. This disorder manifests as pulmonary and vena cava congestion. Heart failure is not an independent disease, but the final stage of several heart diseases. The vast majority of cases of heart failure affect the left side of the heart, and the first manifestation is often pulmonary congestion. Severe pump failure is defined as New York Heart Association (NYHA) classification grade ≥ 3. Target lesion revascularization (TLR) is defined as repeated interventional therapy or bypass surgery within the stent and within 5 mm of the proximal and distal ends of the stent. Malignant arrhythmia includes ventricular tachycardia, ventricular fibrillation, and high atrioventricular block.

### 2.3. Research and Treatment Methods

**Figure 2:**
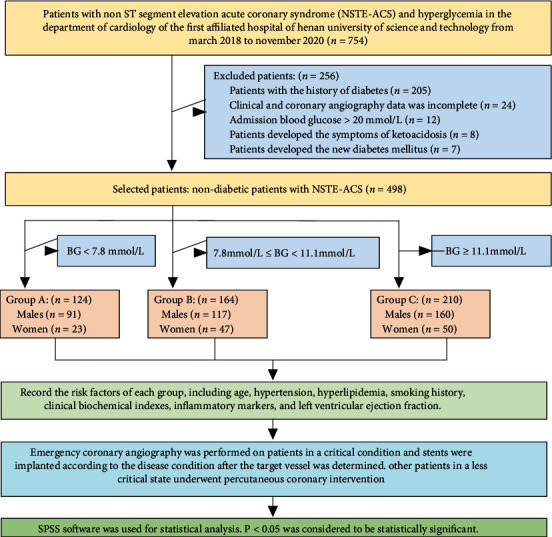
Research scheme.

Patients with suspected ACS underwent an electrocardiogram (ECG) and measurement of the BG level (hexokinase method, Olympus AU400) and myocardial injury markers. ACS was classified by the cardiovascular doctor into unstable angina pectoris, acute ST-segment elevation myocardial infarction, and acute NSTE myocardial infarction based on the ECG and levels of myocardial injury markers. Emergency coronary angiography was performed on patients in a critical condition, such as intractable or recurrent angina pectoris with dynamic ST-segment changes, heart failure, life-threatening arrhythmia, or hemodynamic instability. Stents were implanted in these patients according to the disease condition after the target vessel was determined. Other patients in a less critical state underwent percutaneous coronary intervention (PCI). The research scheme of this paper is shown in Figure 2.

After admission to the Department of Cardiology of the First Affiliated Hospital of Henan University of Science and Technology, the BG level of the patients was checked and ACS was classified into different types. We identified 754 NSTE-ACS with hyperglycemia but excluded 256 patients due to history of diabetes (*n* = 205), incomplete data (*n* = 24), BG level > 20 mmol/L (*n* = 12), symptoms of ketoacidosis (*n* = 8), or new-onset diabetes mellitus (*n* = 7). Finally, 498 NSTE-ACS patients were included in the study.

Based on the BG level, NSTE-ACS patients were divided into three groups: A (BG < 7.8 mmol/L), B (7.8 mmol/L ≤ BG < 11.1 mmol/L), and C (BG ≥ 11.1 mmol/L). The risk factors for ACS were recorded for each group, including age, hypertension, hyperlipidemia, smoking history, clinical biochemical indexes, inflammatory markers, and left ventricular ejection fraction as measured by echocardiography. SPSS software (IBM Corp., Armonk, NY, USA) was used to perform statistical analysis. Differences were considered statistically significant at *p* < 0.05.

### 2.4. Data Quality Control

The quality of the statistical data affects the research accuracy. Data quality control requires scientific and rigorous work. In the context of big data, the quality and efficiency of hospital data should be continuously improved.

All of the departments providing the data were incorporated into the information construction by our hospital to establish an ideal data quality management system. The hospital employees continuously regularly their skills and were familiar with the operation process of the information system. Our hospital regularly monitored the data operation and randomly checked the quality and standardization of the statistical to identify and solve potential problems. This ensured smooth information interaction in the hospital and improved the accuracy of the information. It also provided a solid foundation for the data quality control in our research.

### 2.5. Statistical Methods

SPSS software (version 25; IBM Corp., Armonk, NY, USA) was used to perform statistical analysis. Continuous variables are represented by ($\bar{x}\pm s$), and the standard deviation *s* is expressed as

(1) $$\begin{array}{c} s=\sqrt{\frac{1}{N}\overset{N}{\underset{i=1}{\sum }}{\left(x_{i}-\bar{x}\right)}^{2}}. \end{array}$$

In (1), *s* represents the standard deviation, *x*_*i*_ represents the variable, and $\bar{x}$ represents the average value.

Categorical variables are expressed as numbers or percentages. Means between the multiple groups were compared using one-way ANOVA. The chi-square test was used to measure the difference between the theoretical and actual values, using the following formula:

(2) $$\begin{array}{c} \chi^{2}=\sum \frac{{\left(f_{o}-f_{e}\right)}^{2}}{f_{e}}. \end{array}$$

In (2), *χ*^2^ represents the chi-square value, *f*_*o*_ represents the actual value, and *f*_e_ represents the theoretical value.

Multivariate logistic stepwise regression was used to calculate the odds ratio (OR) for the predictors of in-hospital death and their impact on the outcomes.

## 3. Results

### 3.1. Comparison of Baseline Characteristics of the Three Groups

There was no significant difference among the three groups in terms of age, sex, hypertension, hyperlipidemia, smoking, and history of myocardial infarction (*p* > 0.05 for all). However, there were significant differences among the three groups in the incidences of the multivessel disease, renal insufficiency, Killip grade III/IV, and emergency PCI (*p* < 0.05 for all), as well as the levels of high-sensitivity C-reactive protein (hs-CRP), cardiac troponin T (TnT), and creatine kinase isoenzyme MB (CK-MB) (*p* < 0.05 for all). Comparison of the baseline characteristics of the three groups is shown in Table 1.

### 3.2. Comparison of In-Hospital Outcomes of the Three Groups

**Figure 3:**
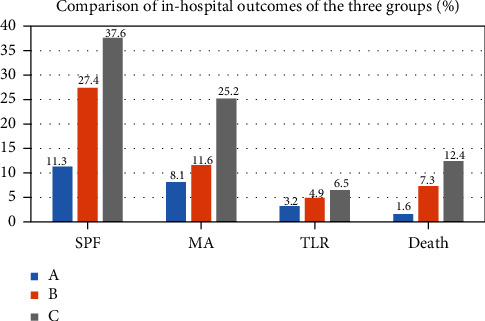
Comparison of the in-hospital outcomes of the three groups.

The incidences of severe pump failure, malignant arrhythmia, and death were significantly higher in groups B and C than in group A (*p* < 0.05). The incidences of severe pump failure, malignant arrhythmia, and death were higher in group C than in group B (*p* < 0.05). Comparison of the in-hospital outcomes of the three groups is shown in Table 2 and Figure 3.

**Figure 4:**
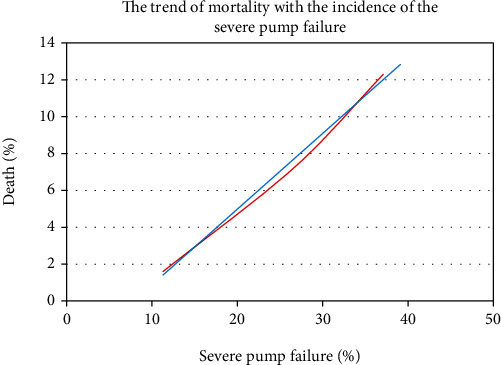
Mortality trend with the incidence of severe pump failure.

Univariate regression analysis of rough data was performed using severe pump failure, malignant arrhythmia, and TLR as independent variables and death as the dependent variable. The percentage of severe pump failure, malignant arrhythmia, and TLR increased significantly with the increase in BG level in the three groups, thereby leading to increased mortality.  Figure 4 shows the mortality trend with the incidence of severe pump failure, showing a linear increase. In Figure 4, red line indicates the univariate regression analysis curve based on the rough data, and blue line indicates the linear prediction trend line for the rough data.

### 3.3. Risk Factors of In-Hospital Death

Logistic regression analysis was performed with in-hospital death as the dependent variable and the previously identified risk factors as independent variables. The results showed that hyperglycemia, age, renal insufficiency, and severe pump failure were risk factors of in-hospital death. Results of the multivariate logistic regression analysis of in-hospital death are shown in Table 3.

## 4. Discussion

Cardiovascular complications are the main cause of death in diabetic patients [7, 8]. Previous studies showed that admission hyperglycemia was an independent risk factor for poor prognosis of ACS patients, irrespective of whether or not they had diabetes [9, 10]. Pasquale first showed that hyperglycemia was associated with adverse outcomes and increased risk of restenosis in ST-elevation myocardial infarction patients without diabetes mellitus [11]. Studies have found that admission hyperglycemia was the greatest risk factor for patients with acute myocardial infarction without diabetes. The 30-day mortality rate of patients without diabetes increased when the admission BG level exceeded 6.1 mmol/L, while the admission BG threshold for the 30-day mortality rate was higher in diabetic patients. Additionally, the increased risk of death associated with high BG level was not limited to known diabetic patients; rather, the mortality rate of patients without diabetes was higher than that of diabetic patients [12, 13]. Yacov et al. reported that admission hyperglycemia was an independent risk factor for acute kidney injury in nondiabetic ST-segment elevation myocardial infarction patients undergoing primary PCI [14]. Stella et al. performed a meta-analysis on the relationship between admission hyperglycemia and myocardial infarct size using cardiovascular magnetic resonance imaging (CMRI) and found that the size of myocardial infarct detected on cardiovascular magnetic resonance positively correlated with admission hyperglycemia in patients with acute myocardial infarction [15]. Ozge et al. reported that elevated admission BG level attenuated the coronary collateral flow in patients with ST-elevation myocardial infarction [16]. Satoshi et al. pointed out that glycemic variability was associated with myocardial damage after PCI in nondiabetic ST-segment elevation myocardial infarction patients [17].

The findings of our study confirmed that the incidence of severe pump failure, malignant arrhythmia, and death in NSTE-ACS patients without diabetes significantly increased with the increase in BG level at admission. The multivariate analysis showed that admission hyperglycemia was a strong risk factor for adverse outcomes in NSTE-ACS patients. The prognosis of NSTE-ACS patients was related to their BG level after treatment. Although intensive hypoglycemia is not required, BG should be controlled within a reasonable range.

It is necessary to determine the pathophysiological mechanism underlying the poor prognosis of ACS patients with hyperglycemia. First, several physiological studies have confirmed that hyperglycemia causes vascular damage and cardiac myocyte death through different molecular mechanisms [18]. Fabio et al. studied hyperglycemia in ACS patients and showed that the collateral circulation decreased and infarct size increased in severely hyperglycemic patients [19]. Fang et al. studied in-hospital peak glycemia for predicting no-reflow phenomenon in diabetic patients with ST-elevation myocardial infarction treated with primary PCI and concluded that spontaneous reperfusion rate of hyperglycemia combined with acute ST-segment elevation myocardial infarction was low [20]. Microvascular dysfunction has also been confirmed in acute myocardial infarction patients with hyperglycemia in the study of Simsek et al. that evaluated the association of acute-to-chronic glycemic ratio and no reflow in patients with ST-segment elevation myocardial infarction undergoing primary PCI [21]. Shock index on admission was associated with coronary slow/no reflow in patients with acute myocardial infarction undergoing emergent PCI. Wang et al. found a higher incidence of no blood flow in patients with hyperglycemia after successful reperfusion [22].

Second, hyperglycemia creates a prothrombotic state. In acutely hyperglycemic mice, the level of tissue plasminogen activator was decreased and the level of plasminogen activation inhibitor was increased. Hyperglycemia in type 2 diabetic patients (abnormal glycemic clamp technique) was associated with increased activity of thromboxane A_2_ (TXA_2_) and von Willebrand factor. Acute hyperglycemia caused fibrinogen *t*_1/2_ to decrease and induced platelet aggregation, thereby increasing the levels of fibrinogen A, prothrombin, and factor VII levels. These changes indicate a prothrombotic state. Third, increased BG level was accompanied by increased vascular inflammatory markers [23, 24]. *In vivo* and *in vitro* studies showed that hyperglycemia was associated with increased hs-CRP, cytokine 26, and tumor necrosis factor *α* levels. In our study, hyperglycemia significantly correlated with increased inflammatory markers, which increased the incidence of adverse cardiac events through the inflammatory response. In addition, higher BG levels correlated with high levels of free fatty acids, greater insulin resistance, and more serious impairment of myocardial glucose utilization in ACS patients. This increased the oxygen consumption, potentially aggravating myocardial ischemia. At the same time, the increased levels of free fatty acids can induce malignant ventricular arrhythmia, which may be one reason for the increased occurrence of in-hospital cardiac arrhythmia with hyperglycemia [25, 26].

Therefore, hyperglycemia has different effects on the prognosis of patients with diabetes or undiagnosed diabetes. Hyperglycemia is more predictive of adverse events in patients with undiagnosed diabetes compared to those with diagnosed diabetes. Although the pathophysiological mechanism underlying this phenomenon is unknown, there are several explanations. Some undiagnosed diabetic patients, especially those with severe hyperglycemia, may be at high risk because they have never been treated for diabetes. In addition, in patients with unknown diabetes and hyperglycemia, when acute myocardial infarction occurs, even if blood glucose was significantly elevated, insulin therapy was rarely used. In view of the possible beneficial effect of insulin on myocardial ischemia, this difference in treatment may explain the different prognosis. Finally, it is possible that similar BG level may represent a more serious condition in unknown diabetic patients. There are still many gaps in understanding the relationship between hyperglycemia and the adverse prognosis. Further studies are needed to confirm whether hyperglycemia is an indicator of high mortality.

## 5. Research Limitations

Some limitations of this study should be considered. First, this is a single-center observational study with a small sample size. Second, in our analysis, intracoronary stenosis was determined on angiography, which may be affected by many factors. In the future, more reliable methods should be used to determine the intracoronary stenosis. Third, unmeasured residual confounding factors that may affect the evaluation of coronary stenosis may exist. Finally, the relatively small study population may theoretically lead to selection bias due to the strict inclusion and exclusion criteria. The conclusions drawn from our findings cannot be extrapolated to the patients excluded from the study.

## Figures and Tables

**Table 1:** Comparison of the baseline characteristics of the three groups.

Group (*n*)	A (*n* = 124)	B (*n* = 164)	C (*n* = 210)	Statistic value	*p* value
Male, *n* (%)	91 (74)	117 (71)	160 (76)	45.217	0.81
Age (year)	65 ± 8	68 ± 7	76 ± 6	19.013^∗^	0.67
Hypertension, *n* (%)	99 (80)	141 (86)	192 (91)	39.571	0.85
Hyperlipidemia, *n* (%)	65 (52)	81 (49)	127 (60)	88.263	0.37
Smoking, *n* (%)	75 (60)	101 (62)	153 (73)	49.215	0.78
Renal inadequacy, *n* (%)	6 (4.8)	18 (11)	28 (13.3)	8.932	0.04
Old myocardial infarction, *n* (%)	12 (9.7)	28 (17.1)	47 (22.4)	13.205	0.27
Triple vessel disease, *n* (%)	43 (34.6)	95 (57.9)	181 (86.2)	68.213	<0.01
Emergency PCI, *n* (%)	81 (65.3)	123 (75)	162 (77.1)	44.312	0.03
Admission blood glucose (mmol/L)	6.2 ± 1.3	9.3 ± 1.5	17.5 ± 4.4	23.981^∗^	<0.01
TnT, *n* (%)	10 (8.1)	63 (38.4)	109 (51.9)	11.251	<0.05
CK-MB, *n* (%)	6 (4.8)	40 (24.4)	95 (45.2)	35.147	<0.05
Killip grading III/IV, *n* (%)	14 (11.3)	43 (26.2)	103 (49.0)	28.102	0.02
Hs-CRP (mg/L)	1.31 ± 0.93	2.13 ± 0.25	3.06 ± 2.61	11.323^∗^	<0.01

Note: ^∗^*F* value. TnT: troponin T; CK-MB: creatine kinase isoenzyme MB; hs-CRP: high-sensitivity C-reactive protein.

**Table 2:** Comparison of in-hospital outcomes of the three groups.

Group	Number	Severe pump failure, *n* (%)	Malignant arrhythmia, *n* (%)	Target lesion revascularization, *n* (%)	Death, *n* (%)
A	124	14 (11.3)	10 (8.1)	4 (3.2)	2 (1.6)
B	164	45 (27.4)^∗^	19 (11.6)^∗^	8 (4.9)^∗^	12 (7.3)^∗^
C	210	79 (37.6)^∗^^△^	53 (25.2)^∗^^△^	14 (6.5)^∗^^△^	26 (12.4)^∗^^△^

Note: ^∗^comparison with group A and ^△^comparison with group B; *p* < 0.05.

**Table 3:** Multivariate logistic regression analysis of in-hospital death.

Item	Odds ratio	95% CI	*p* value
Age	1.03	(0.91, 1.14)	0.23
Hyperglycemia at admission	1.81	(1.26, 2.41)	<0.01
Killip grading III/IV	2.16	(1.03, 3.96)	0.02
Renal insufficiency	1.12	(1.03, 1.21)	0.04

## Data Availability

The data used to support the findings of this study are included within the article.
